# Supplementary material for: Heat adaptation of phage T7 under an extended genetic code
Source: Virus Evol. 2021 Dec 1;7(2):veab100. doi: 10.1093/ve/veab100 (PMC8923235; doi:10.1093/ve/veab100)
Supplement: veab100_Supp [file veab100_supp.zip › Sequencing_Analysis.zip!/Sequencing_Analysis/A1/evidence/JC_57.html]

BRESEQ :: Evidence


| New junction evidence | | | | | | | | | | | |
| --- | --- | --- | --- | --- | --- | --- | --- | --- | --- | --- | --- |
|  | | seq id | position | reads (cov) | reads (cov) | score | skew | freq | annotation | gene | product |
| \* | ? | NC\_001604.1 | = 13277 | 2147 (0.700) | 671 (0.220) | 245/586 | 2.8 | 38.4% | intergenic (–/–) | *–/–* | –/– |
|  | ? | NC\_001604.1 | 14297 = | 53 (0.020) |  |  |  |  | intergenic (–/–) | *–/–* | –/– |

Only 100 of 738 total aligned reads displayed.  

`AGACAAAGGTAAAGCACATGAGGAAGGTCGCCCCGTTTCTATTACTGACCTACGTGGTTCTGGCGCACTACGCCAACTATCTGATACTATTATTGCCCTTGAGCGTAATCAGCAAGGCGATATGCCTAACCTTGTCCTCGTTCGTATTCTCAAGTGCCGCTTTACTGGTGATACTGGTATCGCTGGCTACATGGAATACAACAAGGAAACCGGATGGCTTGAACCATCAAGTTACTCAGGGGAAGAAGAGTCACACTCAGAGTCAACAGACTGGTCCAACGACACTGACTTCTGACAGGATTCTTGA‑‑‑‑‑‑‑‑‑‑‑‑‑‑‑‑‑‑‑‑‑‑‑‑‑‑‑‑‑‑‑‑‑‑‑‑‑‑‑‑‑‑‑‑‑‑‑‑‑‑‑‑‑‑‑‑‑‑‑‑‑‑‑‑‑‑‑‑‑‑‑‑‑‑‑‑‑‑‑‑‑‑‑‑‑‑‑‑‑‑‑‑‑‑‑‑‑‑‑‑‑‑‑‑‑‑‑‑‑‑‑‑‑‑‑‑‑‑‑‑‑‑‑‑‑‑‑‑‑‑‑‑‑‑‑‑‑‑‑‑‑‑‑‑‑‑‑‑‑‑‑‑‑‑‑‑‑‑‑‑‑‑‑‑‑‑‑‑‑‑‑‑‑‑‑‑‑‑‑‑‑‑‑‑‑‑‑‑‑‑‑‑‑‑‑‑‑‑‑‑‑‑‑‑‑‑‑‑‑‑‑‑‑‑‑‑‑‑‑‑‑‑‑‑‑‑‑‑‑‑‑‑‑‑‑‑‑‑‑‑‑‑‑‑‑‑‑‑‑‑‑‑‑‑‑‑‑‑‑‑‑‑‑‑‑‑‑‑‑‑‑‑‑‑‑‑‑‑‑‑‑‑‑‑‑‑‑‑‑‑‑‑‑‑‑‑‑‑‑  >  NC_001604.1/12971‑13277`  
`‑‑‑‑‑‑‑‑‑‑‑‑‑‑‑‑‑‑‑‑‑‑‑‑‑‑‑‑‑‑‑‑‑‑‑‑‑‑‑‑‑‑‑‑‑‑‑‑‑‑‑‑‑‑‑‑‑‑‑‑‑‑‑‑‑‑‑‑‑‑‑‑‑‑‑‑‑‑‑‑‑‑‑‑‑‑‑‑‑‑‑‑‑‑‑‑‑‑‑‑‑‑‑‑‑‑‑‑‑‑‑‑‑‑‑‑‑‑‑‑‑‑‑‑‑‑‑‑‑‑‑‑‑‑‑‑‑‑‑‑‑‑‑‑‑‑‑‑‑‑‑‑‑‑‑‑‑‑‑‑‑‑‑‑‑‑‑‑‑‑‑‑‑‑‑‑‑‑‑‑‑‑‑‑‑‑‑‑‑‑‑‑‑‑‑‑‑‑‑‑‑‑‑‑‑‑‑‑‑‑‑‑‑‑‑‑‑‑‑‑‑‑‑‑‑‑‑‑‑‑‑‑‑‑‑‑‑‑‑‑‑‑‑‑‑‑‑‑‑‑‑‑‑‑‑‑‑‑‑‑‑‑‑‑‑‑‑‑‑‑‑‑‑‑‑‑‑‑‑‑‑‑‑‑‑‑‑‑‑‑‑‑‑‑‑‑‑‑‑‑ttcttgaCAGTTGTTTCATATGAAGAGATTGTTAAGTCACGATAATC‑AATAGGAGAAATCAATATGATCGTTTCTGACATCGAAGCTAACGCCCTCTTAGAGAGCGTCACTAAGTTCCACTGCGGGGTTATCTACGACTACTCCACCGCTGAGTACGTAAGCTACCGTCCGAGTGACTTCGGTGCGTATCTGGATGCGCTGGAAGCCGAGGTTGCACGAGGCGGTCTTATTGTGTTCCACAACGGTCACAAGTATGACGTTCCTGCATTGACCAAACTGGCAAAGTTGCAATTGAACCGAGAGTT  >  NC_001604.1/14297‑14594`  

                                                                                                                                                                                                                                                                                                                                                                                                                                                                                                                                                                                                                                
 `GACAAAGGTAAAGCACATGAGGAAGGTCGCCCCGTTTCTATTACTGACCTACGTGGTTCTGGCGCACTACGCCAACTATCTGATACTATTATTGCCCTTGAGCGTAATCAGCAAGGCGATATGCCTAACCTTGTCCTCGTTCGTATTCTCGAGTGCCGCTTTACTGGTGATACTGGTATCGCTGGCTACATGGAATACAACAAGGAAACCGGATGGCTTGAACCATCAAGTTACTCAGGGGAAGAAGAGTCACACCCAGAGTCAACAGACTGGTCCAACGACACTGACTTCTGACAGGATT                                                                                                                                                                                                                                                                                                                  >  1:210685/1‑301`  
 `GACAAAGGTAAAGCACATGAGGAAGGTCGCCCCGTTTCTATTACTGACCTACGTGGTTCTGGCGCACTACGCCAACTATCTGATACTATTATTGCCCTTGAGCGTAATCAGCAAGGCGATATGCCTAACCTTGTCCTCGTTCGTATTCTCAAGTGCCGCTTTACTGGTGATACTGGTATCGCTGGCTACATGGAATACAACAAGGAAACCGGATGGCTTGAACCATCAAGTTACTCAGGGGAAGAAGAGTCACACTCAGAGTCAACAGACTGGTCCAACGACACTGACTTCTGACAGGATT                                                                                                                                                                                                                                                                                                                  >  1:146675/1‑301`  
 `GACAAAGGTAAAGCACATGAGGAAGGTCGCCCCGTTTCCATTACTGACCTACGTGGTTCTGGCGCACTACGCCAACTATCTGATACTATTATTGCCCTTGAGCGTAATCAGCAAGGCGATAAGCCTAACCTTGTCCTCGTTCGTATTCTCAAGTGCCGCTTTACTGGTGATACTGGTATCGCTGGCTACATGGAATACAACAAGGAAACCGGATGGCTTGAACCATCAAGTTACTCAGGGGAAGAAGAGTCACACTCAGAGTCAACTGAATGGTCCAACGCCACTGACTTCTGACAGGATT                                                                                                                                                                                                                                                                                                                  >  1:274514/1‑301`  
     `AAGGTAAAGCACATGAGGAAGGTCGCCCCGTTTCTATTACTGACCTACGTGGTTCTGGCGCACTACGCCAACTATCTGATACTATTATTGCCCTTGAGCGTAATCAGCAAGGCGATATGCCTAACCTTGTCCTCGTTCGTATTCTCAAGTGCCGCTTTACTGGTGATACTGGTATCGCTGGCTACATGGAATACAACAAGGAAACCGGATGGCTTGAACCATCAAGTTACTCAGGGGAAGAAGAGTCACACTCAGAGTCAACAGACTGGTCCAACTACACTGACTTCTGACAGGATTCTTG                                                                                                                                                                                                                                                                                                              <  1:173794/301‑1`  
      `AGGTAAAGCACATGAGGAAGGTCGCCCCGTTTCTATTACTGACCTACGTGGTTCTGGCGCACTACGCCAACTATCTGATACTATTATTGCCCTTGAGCGTAATCAGCAAGGCGATATGCCTAACCTTGTCCTCGTTCGTATTCTCAAGTGCCGCTTTACTGGTGATACTGGTATCGCTGGCTACATGGAATACAACAAGGAAACCGGATGGCTTGAACCATCAAGTGACTCAGGGGAAGAAGAGTCACACTCAGAGTCAACAGACTGGTCCAACGACACAGACTTCTGACAGGATTCTTGA                                                                                                                                                                                                                                                                                                             <  1:195733/301‑1`  
       `agacagGCACATGAGGAAGGTCGCCCAGTTTCTATTACTGACCTACGTGGTTCTGGCGCACTACGCCAACTATCTGATACTATTATTGCCCTTGAGCGTAATCAGCAAGGCGATATGCCTAACCTTGTCCTCGTTCGTATTCTCAAGTGCCGCTTTACTGGTGATACTAGTATCGCTGGCTACATGGAATACAACAAGGAAACCGGATGGCTTGAACCATCAAGTTACTCAGGGGAAGAAGAGTCACACTCAGAGTCAACAGACTGGTCCAACGACACTGACTTCTGACAGGATTCTTGAC                                                                                                                                                                                                                                                                                                            <  1:230886/295‑1`  
       `GGTAAAGCACATGAGGAAGGTCGCCCCGTTTCTATTACTGACCTACGTGGTTCTGGCGCACTACGCCAACTATCTGATACTATTATTGCCCTTGAGCGTAATCAGCAAGGCGATATGCCTAACCTTGTCCTCGTTCGTATTCTCAAGTGCCGCTTTACTGGTGATACTGGTATCGCTGGCTACATGGAATACAACAAGGAAACCGGATGGCTTGAACCATCAAGTTACTCAGGGGAAGAAGAGTCACACTCAGAGTCAACAGACTGGTCCAACGACACTGACTTCTGACAGGATTCTTGAC                                                                                                                                                                                                                                                                                                            >  1:164971/1‑301`  
              `aACATGAGGAAGGTCG‑CCCGTTTCTATTACTGACCTACGTGGTTCTGGCGCACTACGCCAACTATCTGATACTATTATTGCCCTTGAGCGTAATCAGCAAGGCGATATGCCTAACCTTGTCCTCGTTCGTATTCTCAAGTGCCGCTTTACTGGTGATACTGGTATCGCTGGCTACATGGAATACAACAAGGAAACCGGATGGCTTGAACCATCAAGTTACTCAGGGGAAGAAGAGTCACACTCAGAGTCAACAGACTGGTCCAACGACACTGACTTCTGACAGGATTCTTGACAGTTGTTT                                                                                                                                                                                                                                                                                                    <  1:150440/300‑1`  
              `CACATGAGGAAGGTCGCCCCGTTTCTATTACTGACCTACGTGGTTCTGGCGCACTACGCCAACTATCTTATACTATTATTGCCCTTGAGCGTAATCAGCAAGGCGATATGCCTAACCTTGTCCTCGTACGTATTCTCAAGTGCCGCTTTACTGGTGATACTGGTATCGCTGGCTACATGGAATACAACAAGGAAACCGGATGGCTTGAACCATCAAGTTACTCAGGGGAAGAAGAGTCACACTCAGAGTCAACAGACTGGTCCAACGACACCGACTTCTGACAGGATTCTTGACAGTTGTT                                                                                                                                                                                                                                                                                                     >  1:110205/1‑301`  
                `CATGAGGAAGGTCGCCCCGTTTCTATTACTGACCTACGTGGTTCTGGCGCACTACGCCAACTATCTGATACTATTATTGCCCTTGAGCGTAATCAGCAAGGCGATATGCCTAACCTTGTCCTCGTTCGTATTCTCAAGTGCCGCTTTACTGGTGATACTGGTATCGCTGGCTACATGGAATACAACAAGGAAACCGGATGGCTTGAACCATCAAGTTACTCAGGGGAAGAAGAGTCACACTCAGAGTCAACAGACTGGTCCAACGACACTGACTTCTGACAGGATTCTTGACAGTTGTTTC                                                                                                                                                                                                                                                                                                   >  1:133845/1‑301`  
                   `GAGGAAGGTCTCCCCGTTTCTATTACTGACCTACGTGGTTCTGGCGCACTACGCCAACTATCTGATACTATTATTGCCCTTGAGCGTAATCAGCAAGGCGATATGCCTAACCTTGTCCTCGTTCGTATTCTCAAGTGCCGCTTTACTGGTGATACTGGTATCGCTGGCTACATGGAATACAACAAGGAAACCGGATGGCTTGAACCATCAAGTTACTCAGGGGAAGAAGAGTCACACTCAGAGTCAACAGACTGGTCCAACGACACTGACTTCTGACAGGATTCTTGctgtctcttataca                                                                                                                                                                                                                                                                                                >  1:218513/1‑287`  
                    `AGGAAGGTCGCCCCGTTTCTATTACTGACCTACGTGGTTCTGGCGCACTACGCCAACTATCTGATACTATTATTGCCCTTGAGCGTAATCAGCAAGGCTATATGCCTAACCTTGTCCTCGTTCGTATTCTCAAGTGCCGCTTTACTGGTGATACTGGTATCGCTGGCTACATGGAATACAACAAGGAAACCGGATGGCTTGAACCATCAAGTTACTCAGGGGAAGAAGAGTCACACTCAGAGTCAACAGACTGGTCCAACGACACTGACTTCTGACAGGATTCTTGACAGTTGTTTCATAT                                                                                                                                                                                                                                                                                               <  1:178420/301‑1`  
                        `AGGTCGCCCCGTTTCTATTACTGACCTACGTGGTTCTGTCGCACTACGCCAACTATCTGATACGATTATTGCCCTTGAGCGTAATCAGCAAGGCGATATGCCTAACCTTGTCCTCGTTCGTATTCTCAAGTGCCGCTTTACTGGTGATACTGGTATCGCTGGCTACATGGAATACAACAGGGAAACCGGATGGCTTGAACCATCAAGTTACTCAGGGGAAGAAGAGTCACACTCAGAGTCAACAGACTGGTCCAACGACACTGACTTCTGACAGGATTCTTGACAGTTGTTTCATATGAAG                                                                                                                                                                                                                                                                                           <  1:122926/301‑1`  
                                   `TTTCTATTACTGACCTACGTGGTTCTGTCGCACTACGCCAACTATCTGATACTATTATTTCCCTTTAGCGTAATCAGCAAGTCGCTATGCCTAACCTTGTTCTCGTTCGTATTCTCAAGTGCCGCTTTACTGGTTATTCTTGTATCGCTGGCTACATGCTATTCCACAAGGAAACCGGATGGCTTGAACCATCAAGTTACTCAGGGGAAGAAGAGCTCCACTCAGTGTCAACAGACTTGTCCAACGACACTGACTTCTGACAGGATTCTTGACAGTTGTATCATTTGAATAGATTTTTAtg                                                                                                                                                                                                                                                                                >  1:470716/1‑299`  
                                    `TTCTATTACTGACCTACGTGGTTCTGGCGCACTACGCAAACTATCTGATACTATTATTGCCCTTGAGCGTAATCAGCAAGGCGATATGCCTAACCTTGTCCTCGTTCGTATTCTCAAGTGCCGCTTTACTGGTGATACTGGTATCGCTGGCTACATGGAATACAACAAGGAAACCGGATGGCTTGAACCATCAAGGTACTCAGGGGAAGAAGAGTCACACTCAGAGTCAACAGACTGGTCCAACGACACTGACTTCTGACAGGATTCTTGACAGTTGTTTCATATGCACAGATTGTTAAGT                                                                                                                                                                                                                                                                               >  1:52122/1‑301`  
                                      `CTATTACTGACCTACGTGGTTCTGGCGCACTACGCCAACTATCTGATACTATTATTGCCCTTTAGCGTAATCAGCAAGGCGATATGCCTAACCTTGTCCTCGTTCGTATTCTCAAGTGCCGCTTTACTGGTGATACTGGTATCGCTGGCTACATGGAATACAACAAGGAAACCGGATGGCTTGAACCATCAAGTTACTCAGGGGAAGAAGAGTCACACTCAGAGTCAACAGACTGGTCCAACGACACTGACTTCTGACAGGATTCTTGACAGTTGTTTCATATGAAGAGATTGTTAAGTCA                                                                                                                                                                                                                                                                             >  1:379896/1‑301`  
                                        `ATTACTGACCTACGTGGTTCTGGCGCACTACGCCAACTATCTGATACTATTATTGCCCTTGAGCGTAATCAGCAAGGCGATATGCCTAACCTTGTCCTCGTTCGTATTCTCAAGTGCCGCTTTACTGGTGATACTGGTATCGCTGGCTACATGGAATACAACAAGGAAACCGGATGGCTTGAACCATCAAGTTACTCAGGGGAAGAAGAGTCACACTCAGAGTCAACAGACTGGTCCAACGACACTGACTTCTGACAGGATTCTTGACAGTTGTTTCATATGAAGAGATTGTTAAGTCACG                                                                                                                                                                                                                                                                           >  1:478353/1‑301`  
                                                 `tgtggctcggagatgggtataagagatagATCTGATACTAGTATTGGCCTTGAGTGTAATCAGCAAGGCTATATGCCTAACCTTGTCCTCGTTCGTATTCTAAAGTGCCGCTTTACTTGTGATACTGGTATCGCTGGGTACATGGAATACAACAAGGAAACCGGATGGCTTGAACCATCAAGTTACTCAGGGGAAGAAGCGTCACACTCAGAGTCAACAGACTGGTCCAACGACACTGACTTCTGACAGGATTCTTGACAGTTGTTTCATATGAAGAGATTGTTAAGTCACGATAATC‑AAT                                                                                                                                                                                                                                                                 <  1:40706/272‑1`  
                                                   `ACGTGGTTCTGGCGCACTACGCCAACTATCTGATACTATTATTGCCCTTGAGCGTAATCAGCAAGGCGATATGCCTAACCTTGTCCTCGTTCGTATTCTCAAGTGCCGCTTTACTGGTGATACTGGTATCGCTGGCTACATGGAATACAACAAGGAAACCGGATGGCTTGAACCATCAAGTTACTCAGGGGAAGAAGAGTCACACTCAGAGTCAACAGACTGGTCCAACGACACTGACTTCTGACAGGAATCTTGACAGTTGTTTCATATGAAGAGATTGTTAAGTCACGATAATC‑AATAG                                                                                                                                                                                                                                                               >  1:482213/1‑301`  
                                                    `CGTGGTTCCGCCGCAATACGCCAACTATCTGATAATATGATTGCCCTTGAGCGTAATCAGCAAGGCGATATGCCTAACCTTGTCCTCGTTAGTATTCTCACGTGCCGCTTTACTGGTGATACTGGTATCGCTGGCTACATGGAATACAACAAGGAAACCGGATGGCTTGATCCATAACGTTACTCAGGGGAAGAGAAGTCACAATCAGAGTCAACAGACTTGTCCAGCGACACTGACTTCTGACAGGATTCTTGACAGTGATTTCATATGAAGAGATTGTTAAGTCACGATAATC‑AATAGG                                                                                                                                                                                                                                                              <  1:94506/301‑1`  
                                                        `GTTCTGGCGCACTACGCCAACTATCTGATACTATTATTGCCCTTGAGCGTAATCAGCAAGGCGATATGCCTAACCTTGTCCTCGTTCGTATTCTCAAGTGCCGCTTTACTGGTGATACTGGTATCGATGGCTACATGGAATACAACAAGGAAACCGGATGGCTTGAACCATCAAGTTACTCAGGGGAAGAAGAGTCACACTCAGAGTCAACAGACTGGTCCAACGACACTGACTTCTGACAGGATTCTTGACAGTTGTTTCATATGAAGAGATTGTTAAGTCACGATAATC‑AATAGGAGct                                                                                                                                                                                                                                                          >  1:346148/1‑299`  
                                                                `gacagACGCCAACTATCTGATACTATTATTGCCCTTGAGCGTAATCAGCAAGGCGATATGCCTAACCTTGTCCTCGTTCGTATTCTCAAGTGCCGCTTTACTGGTGATACTGGTATCGCTGGCTACATGGAATACAACAAGGAAACCGGATGGCTTGAACCATCAAGTTACTCAGGGGAAGAAGAGTCACACTCAGAGTCAACAGACTGGTCCAACGACACTGACTTCTGACAGGATTCTTGACAGTTGTTTCATATGAAGAGATTGTTAAGTCACGATAATC‑AATAGGAGAAATCAATAT                                                                                                                                                                                                                                                  <  1:25112/296‑1`  
                                                                   `CTACGCCAACTATCTGATACTATTATTGCCCTTGAGCGTAATCAGCAAGGCGATATGCCTAACCTTGTCCTCGTTCGTATTCTCAAGTGCCGCTTTACTGGTGATACTGGTATCGCTGGCTACATGGAATACAACAAGTAAACCGGATGGCTTGAACCATCAAGTTACTCAGGGTAAGAAGAGTCACCCTCAGAGTCAACAGACTGGTCCAACGACACTGACTACTGACAGGATTCTTGACAGTTGTTTCATATGAAGAGATTGTTAAGTCACGATAATC‑AATAGGAGAAATCAATATGAT                                                                                                                                                                                                                                               >  1:183575/1‑301`  
                                                                   `CTACGCCAACTATCTGATACTATTATTGCCCTTGAGCGTAATCAGCAAGGCGATATGCCTAACCTTGTCCTCGTTCGTATTCTCAAGTGCCGCTTTACTGGTGATACTGGTATCGCTGGCTACATGGAATACAACAAGGAAACCGGATGGCTTGAACCATCAAGTTACTCAGGGGAAGAAGGGTCACACTCAGAGTCAACAGACTGGTCCAACGACACTTACTTCTGACAGGTTTCTTGACAGTTGTTTCATATGAAGAGATTGTTAAGTCACGATAATA‑AATAGGAGctgtatcttatac                                                                                                                                                                                                                                               >  1:340687/1‑288`  
                                                                   `CTACGCCAACTATCTGATACTATTATTGCCCTTGAGCGTAATCAGCAAGGCGATATGCCTAACCTTGTCCTCGTTCGTATTCTCAAGTGCCGCTTTACTGGTGATACTGGTATCGCTGGCTACATGGAATACAACAAGGAAACCGGATGGCTTGAACCATCAAGTTACTCAGGGGAAGAAGAGTCACACTAAGAGTCAACAGACTGGTCCAACGACACTGACTTCTGACAGGATTCTTGACAGTTGTTTCATATGAAGAGATTGTCAAGTCACGATAATC‑AATAGGAGAAATCAATATGAT                                                                                                                                                                                                                                               <  1:336621/301‑1`  
                                                                           `gagtataagataaaagATTGCCCTGGAGCGTAATACGAAAGGCGATATGCCTAACCTTGTCCTCGTTCGTATTATAAAGTGCCGCTTTAATGGGGATACTGGTATCGCTGGCTACAGGGAATACAACAAGGAAACCGGATGGCTTGAACCATCAAGTTACTCAGGGGAATAAGAGTCACACTCAGAGTCAACAGACTGGTCAAACGACACTGACTTCTGACAGGTTTCTTGACAGTTGTTTCATATGAAGAGATTGTTAAGTCACGATAATC‑AATAGGAGAAATCAATATGATCGTTTCTG                                                                                                                                                                                                                                       <  1:495538/285‑1`  
                                                                            `CTATCTGATACTATTATTGCCCTTGAGCGTAATCAGCAAGGCGATATGCCTAACCTTGTCCTCGTTCGTATTCTCAAGTGCCGCTTTACTGGTGATACTGGTATCGATGGCTACATGGAATACAACAAGGAAACCGGATGGCTTGAACCATCAAGTTACTCCGGGGAAGAAGAGTCACACTCAGAGTCAACAGAATGGTCCAACGACACTGACTTCTGACAGGATTCTTTACAGTTGTTTCATATGAAGACATTGTTCAGTCACGACAATC‑AATAGGAGAAATCAATATGcactttctctt                                                                                                                                                                                                                                      >  1:312106/1‑290`  
                                                                              `ATCTGATACTATTATTGCCCTTGAGCGTAATCAGCAAGGCGATATGCCTAACCTTGTCCTCGTTCGGATTCTCAAGTGCCGCTTTACTGGTGATACTGGTATCGCTGGCTACATGGAATACAACAAGGAAACCGGATGGCTTGAACCATCAAGTTACTCAGGGGAAGAAGAGTCACACTCAGAGTCAACAGACTGGTCCAACGACACTGACTTCTGACAGGATTCTTGACAGTTGTTTCATATGAAGAGATTGTTAAGTCACGATAATC‑AATAGGATAAATCAATATGATCGTTTCTGACA                                                                                                                                                                                                                                    <  1:443347/301‑1`  
                                                                                     `ACTATTATTGCCATTGAGCGTAATCAGCAAGGCGATATGCCTAACCTTGTCATCGTTAGTATTATCAAGTGCCGCTGTACTGGTGATACTGGTATCGCTTGCTACATGGAATACAAAAAGGAACCCGTATGGCTTGAACCATCAAGTTACTCAGGGGAAGAAGAGTCACACTCAGAGTCAACATACTGGTCCAACGACACTTACTTCTGACAGGATTCTTGACAGTTGTTTCATATGAAGAGATTGTTAAGTCACGATAATC‑AATAGGAGAAATCAATATGATCGTTTCTGACATCGAAGC                                                                                                                                                                                                                             <  1:306621/301‑1`  
                                                                                        `ATTATTGCCCTTGAGCGTAATCAGCAAGGCGATATGCCTAACCTTGTCCTCGTTCGTATTCTCAAGTGCCGCTTTACTGGTGATACTGGTATCGCTGGCTACATGGAATACAACAAGGAAACCGGATGGCTTGAACCATCAAGTTACTCAGGGGAAGAAGAGTCACACTCAGAGTCAACAGACTGGTCCAACGACACTGACTTCTGACAGGATTCTTGACAGTTGTTTCATATGAAGAGATTGTTAAGTCACGATAATC‑AATAGGAGAAATCAATATGATCGTTTCTGACATCGAAGCTAA                                                                                                                                                                                                                          >  1:107422/1‑301`  
                                                                                        `ATTATTGCCCTTGAGCGTAATCAGCAAGGCGATATGCCTAACCTTGTCCTCGTTCGTATTCTCAAGTGCCGCTTTACTGGTGATACTGGTATCACTGGCTACATGGAATACAACAAGGAAACCGGATGGCTTGAACCATCAAGTTACTCAGGGGAAGAAGAGTCACACTAAGAGTCAACAGACTGGTCCAACGACACTGACTTCTGACAGGATTCTTGACAGTTGTTTCATATGAAGAGATTGTTAAGTCACGATAATC‑AATAGGAGAAACCATTATGATCGTTTCTGACATCGAAGCTAA                                                                                                                                                                                                                          >  1:384393/1‑301`  
                                                                                         `TTATTGCCCTTGAGCGTAATCAGCAAGGCGATATGCCTAACCTTGTCCTCGTTCGTATTCTCAAGTTCCGCTTTACTGGTGATACTGGTATCGCTGGCTACATGGAATACAACAAGGAAACCGGATGGCTTGAACCATCAAGTTACTCAGGGGAAGAAGAGTCACCCTCAGAGTCAACAGACTGGTCCAACGACACTGACTTCTGACAGGATTCTTGACAGTTGTTTCATATGAAGAGATTGTTAAGTCACGATAATC‑AATAGGAGAAATCAATATGATCGTTTTTGAAATCGAAGCTAAC                                                                                                                                                                                                                         >  1:157988/1‑301`  
                                                                                                     `gaaacagTCAGCAAGGCGATATGCCTAACCTTGTCCTCGTTCGTATTCTCAAGTGCCGCTTTACTGGTGATACTGGTATCGCTGGCTAAATGGAATACAACAAGGAAACCGGATGGTTTGAACCATCAAGTTACTCAGGGGAAGAAGAGTCACACTCAGAGTAAACAGACTGGTCCAACGACACTGACTTCTGACAGGATTCTTGACAGTTGTTTCATATGAAGAGATTGTTAAGTCACGATAATC‑AATAGGAGAAATCAATATGATAGTTTCTGACATCGAAGCTAACGCCCTCTTAGAG                                                                                                                                                                                                             <  1:265716/294‑1`  
                                                                                                     `atCGTAATCAGCAAGGCGATCTGCCTAACCATGTCCTCGTTTGTATTCTCAAGTGACGTTTTACTGGTGATACTGGTATCGCTGGCTACATGGAATACAACAAGGAAACCGGATGGCTTGAAACATAAAGTTAATCAGGGGAAGAAGAGTCACACTCAGAGTCAACAGACTGGTCCAACGACACTGACTTCTGACAGGATTCTTGACAGTTGTTTCATATGAAGAGATTGTTAAGTCACGATAATC‑AATAGGAGAAATCAATATGATCGTTTCTGACATCGAAGCTAACGCCCTCTTAGAG                                                                                                                                                                                                             <  1:42235/299‑1`  
                                                                                                     `AGCTTAATCAGAAAGTCGATATGCCTAACCTTGTCCTCGTTCGTATTCTCAAGTGCCGATTTACTGGTGATACTGGTATCGCTGGCTACATGGAATACAACAAGGAAACCGGATGGCTTGAACCATCAAGTTACTCAGGGGAAGAAGAGTCACACTCAGAGTAAACAGACTGGTCCAACGACACTGACTTCTGACAGGATTCTTGACAGTTGTTTCATATGAAGAGATTGTTAAGTCACGATAATC‑AATAGGAGAAATCAATATGATCGTTTCTGACATCGAAGCTAACGCCCTCTTAGAG                                                                                                                                                                                                             <  1:86958/301‑1`  
                                                                                                     `AGCGTAATCAGCAAGGCGATATGCCTAACCTTGTCCTCGTTCGTATTCTCAAGTGCCGCTTTACTGGTGATACTGGTATCGCTGGCTACATGGAATACAACAAGGAAACCGGATGGCTTGAACCATCAAGTTACTCAGGGGAAGAAGAGTCACACTCAGAGTCAACAGACTGGTCCAACGACACTGACTTCTGACAGGATTCTTGACAGTTGTTTCATATGAAGAGATTGTTAAGTCACGATAATC‑AATAGGAGAAATCAATATGATCGTTTCTGACATCGAAGCTAACGCCCTCTTAGAG                                                                                                                                                                                                             <  1:439800/301‑1`  
                                                                                                       `aGTAATCAGCAAGGCGATATGCCTAACCTTGTCCTCGTTCGTATTCTCAAGTGCCGCTTTACTGGTGATACTGGTATCGCTGGCTACATGGAATACAACAAGGAAACCGGATGGCTTGAACCATCAAGTTACTCAGGGGAAGAAGAGTCACACTCAGAGTCAACAGACTGGTCCAACGACACTGACTTCTGACAGGATTCTTGACAGTTGTTTCATATGAAGAGATTGTTAAGTCACGATAATC‑AATAGGAGAAATCAATATGATCGTTTCTGACATCGAAGCTAACGCCCTCTTAGAGAG                                                                                                                                                                                                           <  1:269018/300‑1`  
                                                                                                             `CAGCAAGGCGATATGCCTAACCTTGTCCTCGTTCGTATTCTCAAGTGCCGCTTTACTGGTGATACTGGTATCGCTGGCTACATGGAATACAACAAGGAAACCGGATGGCTTGAACCATCAAGTTACTCAGGGGAAGAAGAGTCACACTCAGAGTCAACAGACTGGTACAACGACACTGACTTCTGACAGGATTCTTGACAGTTGTTTCATATGAAGAGATTGTTAAGTCACGATAATC‑AATAGGCTAAATCAATATAATCGTTTCTGACATCGACGCTAACGCCCTCTTAGAGAGAGTCAC                                                                                                                                                                                                     >  1:75568/1‑301`  
                                                                                                               `GCAAGGCGATATGCCTAACCTTGTCCTCGTTCGTATTCTCAAGTGCCGTTTTACTGGTGATACTGGTATCGCTGGCTACATGGAATACAACAAGGAAACCGGATGGCTTGAACCATCAAGTTACTCAGGGGAAGAAGAGTCACACTCAGAGTCAACAGACTGGTCCAACGACACTGACTTCTGACAGGATTCTTGACAGTTGTTTCATATGAAGAGATTGTTAAGTCACGATAATC‑AATAGGAGAAATCAATATGATCGTTTCTGACATCGAAGCTAACGCCCTCTTAGAGAGCGTCACTA                                                                                                                                                                                                   <  1:202684/301‑1`  
                                                                                                                    `GCGATATGCCTAACCTTGTCCTCGTTCGTATTCTCAAGTGCCGCTTTACTGGTGATACTGGTATCGCTGGCTACATGGAATACAACAAGGAAACCGGATGGCTTGAACCATCAAGTTACTCAGGGGAAGAAGAGTCACACTCAGAGTCAACAGACTGGTCCAACGACACTGACTTCTGACAGGATTCTTGACAGTTGTTTCATATGAAGAGATTGTTAAGTCACGATAATC‑AATAGGAGAAATCAATATGATCGTTTCTGACATCGAAGCTAACGCCCTCTTAGAGctgtctcttatacac                                                                                                                                                                                              >  1:235062/1‑286`  
                                                                                                                     `gGATATGCCTAACCTTGTCCTAGTTAGTATTCGCAAGTGCCGCTTTACTGGTGATACTGGTATCGCGGGCTACATGGAATACAACAAGGAAACCGGATGGCTTGAACCATCAAGTTACTCAGGGGAAGAAGAGTCACACTCAGAGTCAACAGACTGGTACAACGACACTGACTTCTGACAGGATTCTTGACAGTTGTTTCATATGAAGAGAGTGTTAAGTCACGATAATC‑AATAGGAGAAATCAATATGATCGTTTCTGACATCGAAGCTAACGCCCTCTTAGAGAGCGTCACTAAGTTCC                                                                                                                                                                                             <  1:162367/300‑1`  
                                                                                                                         `ATGCCTAACCTTGTCCTCGTTCGTATTCTCAAGTGCCGCTTTACTGGTGATACTGGTATCGCTGGCTACATGGAATACAACAAGGAAACCGGATGGCTTGAACCATCAAGTTACTCAGGGGAAGAAGAGTCACACTCAGAGTCAACAGACTGGTCCAACGACACTGACTTCTGACAGGATTCTTGACAGTTGTTTCATATGAAGAGATTGTTAAGTCACGATAATC‑AATAGGAGAAATCAATATGATCGTTTCTGACATCGAAGCTAACGCCCTATTAGAGAGCGTCACTAAGTTCCCCTG                                                                                                                                                                                         >  1:32754/1‑301`  
                                                                                                                           `GCCTAACCTTGTCCTCGTTCGTATTCTCAAGTGCCGCTTTACTGGTGATACTGGTATCGCTGGCTACATGGAATACAACAAGGAAACCGGATGGCTTGAACCATCAAGTTACTCAGGGGAAGAAGAGTCACACTCAGAGTAAACAGACTGGTCCAACGACACTGACTTCTGACAGGATTCTTGACAGTTGTTTCATATGAAGAGATTGTTAAGTCACGATAATC‑AATAGGAGAAATCAATATGATCGTTTCTGACATCGAAGATAACGCCCTCTTAGAGAGCGTCACTAAGTTCCACTGCG                                                                                                                                                                                       >  1:415373/1‑301`  
                                                                                                                           `GCCTAACCTTGTCCTCGTTCGTATTCTCAAGTGCCGCTTTACTGGTGATACTGGTATCGCTGGCTACATGGAATACAACAAGGAAACCGGATGGCTTGAACCATCAAGTTACTCAGGGGAAGAAGAGTCACACTAAGAGTCAACAGACTGGTCCAACGACACTGACTTC‑‑‑‑‑‑‑‑‑‑‑‑TGACAGTTGTTTCATATGAAGAGATTGTTAAGTCACGATAATC‑AATAGGAGAAATCAATATGATCGTTTCTGACATCGAAGCTAACGCCCTCTTAGAGAGCGTCACTAAGTTCCACTGCGGGGTTATCTACG                                                                                                                                                                           >  1:58079/1‑301`  
                                                                                                                                `ACCTTGTCCTCGTTCGTATTCTCAAGTGCCGCTTTACTGGTGATACTGGTATCGCTGGCTACATGGAATACAACAAGGAAACCGGATGGCTTGAACCATCAAGTTACTCAGTGGAATAAGAGTCACACTCAGAGTCAACAGACTGGTCCAACGACACTGCCTTCTTACAGGATTCTTGACAGTTGTTTCATATGAAGAGATTGTTAAGTCACGATAATC‑AATAGGAGAAATCAATATGATCGTTTCTGACATCGAAGCTAACGCCCTCTTAGAGAGCGTCCCTAAGTTCCACTGCGGGGTT                                                                                                                                                                                  >  1:271026/1‑301`  
                                                                                                                                          `CGTTCGTATTCTCAAGTGCCGCTTTACTGGTGATACTGGTATCGCTGGCTACATGGAATACAACAAGGAAACCGGATGGCTTGAACCATCAAGTTACTCAGGGGAAGAAGAGTCACACTCAGAGTCAACAGACTGGTCCAACGACACTGACTTCTGACAGGATTCTTGACAGTTGTTTCATATGAAGAGATTGTTAAGTCACGATAATC‑AATAGGAGAAATCAATATGATCGTTTCTGACATCGAAGCTAACGCACTCTTAGAGAGCGTAACTAAGTTCCACTGCGGGGTTATCTACGACT                                                                                                                                                                        >  1:190811/1‑301`  
                                                                                                                                              `CGTATTCTCAAGTGCCGCTTTACTGGTGATACTGGTATCGCTGGCTACATGGAATACAACAAGGAAACCGGATGGCTTGAACCATCAAGTTACTCAGGGGAAGAAGAGTCACACTCAGAGTCAACAGACTGGTCCAACGACACTGACTTCTGACAGGATTCTTGACAGTTGTTTCATATGAAGAGATTGTTAAGTCACGATAATC‑AATAGGAGAAATCAATATGATCGTTTCTGACATCGAAGCTAACGCCCTCTTAGAGAGCGTCACTAAGTTCCACTGCGGGGTTATCTACGACTACTC                                                                                                                                                                    <  1:42952/301‑1`  
                                                                                                                                               `ttgtataagtgccagGCTTTACTGGTGATACTGGTATCGCGGGCTACATGGAATACAACAAGGAAACCGGATGGCGTGAACCATCAAGTTACTCAGGGGAAGAAGAGTCACACTCAGAGTAAACAGACTGGTCCAACGACACTGACATCTGACAGGATTCTTGACAGTTGTTTCATATGAAGAGATTGTTAAGTCACGATAATC‑AATAGGAGAAATCAATATGATAGTTTCTGACATCGAAGCTAACGCCCTCTTAGAGAGCGTCACTAAGTTCCACTGCGGGGTTATCTACGACTACTCC                                                                                                                                                                   <  1:16756/286‑1`  
                                                                                                                                                 `ATTCTCAAGTGCCGCTTTACGGGTGATACTGGTATCGCTGGCTACATGGAATACAACAAGGAAACCGGATGGCTTGAACCATCAAGTTACTCAGGGGAAGAAGAGTCACACTCAGAGTCAACAGACTGGTCCAACGACACTGACTTCTGACAGGATTCTTGACAGTTGTTTCATATGAAGAGATTGTTAAGTCACGATAATC‑AATAGGAGAAATCAATATGATCGTTTCTGACATCGAAGCTAACGCCCTCTTAGAGAGCGTCACTAAGTTCCACTGCGGGGTTATCTACGACTACTACAC                                                                                                                                                                 <  1:138389/301‑1`  
                                                                                                                                                    `gtgtataagagacagACTGGTGATACTGGTATCGATGGCTACATGGAATACAACAAGGAAACCGGATGGCTTGAACCATCAAGTTACTCAGGGGAAGCAGAGTCACACTCAGAGTCAACAGACTGGTCCAACGACACTGACTTCTGACAGGATTCTTGACAGTTGTTTCATATGAAGAGATTGTTAAGTCACGATAATC‑AATAGGAGAAATCAATATGATCGTTTCTGACATCGAAGCTAACGCCCTCTTAGAGAGCGTCACTAAGTTCCACTGCGGGGTTATCTACGACTACTACACCGC                                                                                                                                                              <  1:12947/286‑1`  
                                                                                                                                                    `CTCAAGTGCCGCTTTACTGGTGATACTGGTATCGCTGGCTACATGGAATACAACAAGGAAACCGGATGGCTTGAACCATCAAGTTACTCAGGGGAAGAAGAGTCACACTCAGAGTCAACAGACTGGTCCAACGACACTGACTTCTGACAGGATTCTTGACAGTTGTTTCATATGAAGAGAGTGTTAAGTCACGATAATC‑AATAGGAGAAATCAATATGATCGTTTCTGACATCGAAGCTAACGCCCTCTTAGAGAGCGTCACTAAGTTCCACTGCGGGGTTATCTACGACTACTCCACCGC                                                                                                                                                              <  1:463000/301‑1`  
                                                                                                                                                           `GCCGCGTTACTGGTGATACTGGTATCGCTTGCTACATGGAATACAACAAGGAAACCGGATGGCTTGAAACATCAAGTTACTCAGGGGAAGAAGAGTCACACTAAGAGTAAACAGACTGGTCCAACGACACTGACTTCTGACAGGATTCTTGACAGTTGTTTCATATGAAGAGATTGTTAAGTCACGATAATC‑AATAGGAGAAATCAATATGATCGTTTCTGACATCGAAGCTAACGCCCTCTTAGAGAGCGTCACTAAGTTCCACTGCGGGGTTATCTACGACTACTCCACCGCTGAGTAC                                                                                                                                                       <  1:368181/301‑1`  
                                                                                                                                                              `GCTTGACTGGTGATACTGGTATCGCTGGCTACATGGAATACAACAAGGAAACCGGATGACTTGAACCATCAAGTTAATCATGGGAAGAAGAGGCACACTCAGAGTCAACAGACTGGTCCAACTACACTGACTTCTGACAGGATTCTTGACAGTTGTTTCATATGAATAGATTGTTAAGTCACGATAATC‑AATAGGAGAAATCAATATGATCGTTTCTGACATCGAAGCTAACGCCCTCTTAGAGAGCGTCACTAAGTTCCACTGCGGGGTTATCTACGACTACTCCACCGCTGAGTACGTA                                                                                                                                                    <  1:91447/301‑1`  
                                                                                                                                                                `acagCTGGTGATACTGGTATCGCTGGCTACATGGAATAAAACAAGGAAACCGGATGGCTTGAACCATCAAGTTACTCAGGGGAAGAAGAGTCACACTCAGAGTCAACAGACTGGTCCAACGACACTGACTTCTGACAGGATTCTTGACAGTTGTTTCATATGAAGAGATTGTTAAGTCACGATAATC‑AATAGGAGAAATCAATATGATCGTTTCTGACATCGAAGCTAACGCCCTCTTAGAGAGCGTCACTAAGTTCCACTGCGGGGTTATCTACGACTACTCCACCGCTGAGTACGTAAG                                                                                                                                                  <  1:424600/297‑1`  
                                                                                                                                                                `TTTACGGGGGATACTGGTATCGATGGCTACAGGGAATACAACAAGGAAAGCGGATGGCTTGAACCATCAAGTTACTCAGGGGAAGAAGAGTCACACTCATAGTCAACAGACTGGTCCAACGACACTGACTTCTGACAGGATTCTTGACAGTTGTTTCATATGAAGAGATAGTTAAGTCACGATAATC‑AATAGGAGAAATCAATATGATCGTTTCTGACATCGAAGCTAACGCCCTCTTAGAGAGCGTCACTAAGTTCCACTGCGGGGTTATCTACGACTACTCCACCGCTGAGTACGTAAG                                                                                                                                                  <  1:446158/301‑1`  
                                                                                                                                                                       `ggctcggagatgtgtataagagacaGGAATACAACAAGGAAACCGGATGGCTTGAACCATCAAGTTACTCAGGGGAAGAAGAGTCACACTCAGAGTCAACAGACTGGTCCAACGACACTGACTTC‑‑‑‑‑‑‑‑‑‑‑‑TGACAGTTGTTTCATATGAAGAGATTGTTAAGTCACGATAATC‑AATAGGAGAAATCAATATGATCGTTTCTGACATCGAAGCTAACGCCCTCTTAGAGAGCGTCACTAAGTTCCACTGCGGGGTTATCTACGACTACTCCACCGCTGAGTACGTAAGCTACCGTCCGAGTGACTTC                                                                                                                               <  1:475036/276‑1`  
                                                                                                                                                                           `TACTGGTATCGCTGGCTACATGGAATACAACAAGGAAACCGGATGGCTTGAACCATCAAGTTACTCAGGGGAAGAAGAGTCACACTCAGAGTCAACAGACTGGTCCAACGACACTGACTTCTGACAGGATTCTTGACAGTTGTTTCATATGAAGAGATTGTTAAGTCACGATAATC‑AATAGGAGAAATCAATATGATCGTTTCTGACATCGAAGCTAACGCCCTCTTAGAGAGCGTCACTAAGTTCCACTGCGGGGTTATCTACGACTACTCCACCGCTGAGTACGTAAGCTACCGTCCtg                                                                                                                                       >  1:8051/1‑299`  
                                                                                                                                                                                        `GGCTACATGGGATACAACAAGGAAACCGGATGGCTTGAACCATCAAGTTACTCAGGGGAAGAAGAGTCACACTCAGAGTCAACAGACTGGTCCAACGACACTGACTTCTGACAGGATTCTTGACAGTTGTTTCATATGAAGAGATTGTTAAGTCACGATAATC‑AATAGGAGAAATCAATATGATCGTTTCTGACATCGAAGCTAACGCCCTCTTAGAGAGCGTCACTAAGTTCCACTGCGGGGTTATCTACGACTACTCCACCGCTGAGTACGTAAGCTACCGTCCGAGTGACTTCGGTGC                                                                                                                          >  1:89653/1‑301`  
                                                                                                                                                                                        `GGCTACATGGAATACAACAAGGAAACCGGATGGCTTGAACCATCAAGTTACTCAGGGGAAGAAGAGTCACACTCAGAGTCAACAGACTGGTCCAACGACACTGACTTCTGACAGGATTCTTGACAGTTGTTTCATATGAAGAGATTGTTAAGTCACGATAATC‑AATAGGAGAAATCAATATGATCGTTTCTGACATCGAAGCTAACGCCATCTTAGAGAGCGTCACTAAGTTCCACTGCGGGGTTATCTACGACTACTCCACCGCTGAGTACGTAAGCTACCGTCCGAGTGACTTCGGTGC                                                                                                                          >  1:373688/1‑301`  
                                                                                                                                                                                              `ATGGAATACAACAAGGAAACCGGATGGCTTGAACCATCAAGTTACTCAGGGGAAGAAGAGTCACACTCAGAGTCAACAGACTGGTCCAACGACACTGACTTCTGCCAGGATTCTTGACAGTTGTTTCATATGAAGAGATTGTTAAGTCACGATAATC‑AATAGGAGAAATCAATATGATCGTTTCTGACATCGAAGCTAACGCCCTCTTAGAGAGCTTCACTAAGTTCCACTGCGGGGTTATCTACGACTACTCCACCGCTGAGTACTTACGCTACCGTCCGAGTTACTTCGGTGCGTATCT                                                                                                                    >  1:48727/1‑301`  
                                                                                                                                                                                               `TGGAATACAACAAGGAAACCGGATGGCTTGAACCATCAAGTTAGTCAGGGGAAGAAGAGTCACACTCAGAGTCAACAGACTGGTCCAACGACACTGACTTCTGACAGGATTCTTGACAGTTGTTTCATATGAAGAGATTGTTAAGTCACGATAATC‑AATAGGAGAAATCAATATGATCGTTTCTGACATCGAAGCTAACGCCCTCTTAGAGAGCGTCACTAAGTTCCACTGCGGGGTTATCTACGACTACTCCACCGCTGAGTACGTAAGCTACCGTCCGAGTGACTTCGGTGCGTATCTG                                                                                                                   <  1:246298/301‑1`  
                                                                                                                                                                                                     `ACAACAAGGAAACCGGATGGCTTGAACCATCAAGTTACTCAGGGGAAGAAGAGTCACACTCAGAGTCAACAGACTGGTACAACGACACTGACTTCTGAGAGGATTCTTGACAGTTGTTTCATATGAAGAGATTGTTAAGTCACGATAATC‑AATAGGAGAAATCAATATGATCGTTTCTGATATCGAAGCTAACGCCCTCTTAGAGAGCGTCACTAAGTGCCACTGCTGGGGTATCTACGATTACTCCAACGCTGAGTGCGTAAGCTAGCGTCCGAGCGCCTTCGGTGCGTATCTGTATGCG                                                                                                             >  1:410942/1‑301`  
                                                                                                                                                                                                             `GAAACCGGATGGCTTGAACCATCAAGTTACTCAGGGGAAGAAGAGTCACACTCAGAGTCAACAGACTGGTCCGACGACACTGACTTCTGACAGGATTCTTGACAGTTGTTTCATATGAAGAGATTGTTAAGTCACGATAATC‑AATAGGAGAAATCAATATGATCGTTTCTGACATCGAAGCTAACGCCCTCTTAGAGAGCGTCACTAAGTTCCACTGCGGGGTTATCTACGACTACTCCACCGCTGAGTACGTAAGCTACCGTCCGAGTGACTTCGGTGCGTATCTGGATGCGCTGGAAGC                                                                                                     >  1:262912/1‑301`  
                                                                                                                                                                                                                           `TGAACCATCAAGTTACTCAGGGGAAGAATAGTAACACTCAGAGTCAACAGACTGGTCCAACGACACTGACTTC‑‑‑‑‑‑‑‑‑‑‑‑TGACAGTTGTTTCAGATGAAGAGATTGTTAAGTCACGATAATA‑AATAGGAGAAATCAATATGATCGTTTCTGACATCGAAGCTAACGCCCTCTTAGAGAGCGTCACTAAGTTCCACTGCGGGGTTATCTACGACTACTCCACCGATGAGTACGTAAGCTACCGTCCGAGTGACTTCGGTGCGTATCTGGATGCGCTGGAAGCCGAGGTTGCACGAGGCGGTCTTATTG                                                                           <  1:83403/301‑1`  
                                                                                                                                                                                                                                    `AAGTTACTCAGGGGAAGAAGAGTCACACTCAGAGTCAACATACTGGTCCAACGACACTGACTTCTGACAGGATTCTTGACAGTTGTTTCATATGAAGAGATTGTTAAGTCACGATAATC‑AATAGGAGAAATCAATATGATCGTTTCTGACATCGAAGCTAACGCCCTCTTAGAGAGCGTCACTAAGCTCCACTGCGGGGTTATCTACGACTACTCCACCGCTGAGTACGTAAGCTACCGTCCGAGTGACTTCGGTGCGTATCTGGATGCGCTGGAAGCCGAGGTTGCACGAGGCGGTCTTt                                                                              >  1:397365/1‑300`  
                                                                                                                                                                                                                                       `tttCTCAGGTGAAGACGAGTCACACTCAGAGTCAACATACTGTTCCAACGACACTGACTTCTGACAGGTTGCTTGACAGTTGTTTCATATGAAGAGTTTGTTAAGTCACGATAATC‑AATAGGGGAAATCAATATGATTGTTTCTGACGTCGAAGCTAACGCCCTCTTAGAGAGCGTCACTAAGTTCAACTGCGGGGTTATCTACGACTACTCCACCGCTGAGTACGTAAGCTACCGTCCGAGTGACTTCGGTGCGTATCTGGATGCGCTGGAAGCCGAGGTTGCACGAGGCGGTCTTATTG                                                                           <  1:354359/298‑1`  
                                                                                                                                                                                                                                       `gtataacagatagGAAGAGTAACACTCAGAGTCAACATACTGGTCCAACGACACTGACTTCTGACAGGATTCTTGACAGTTGTTTCATATGAAGAGATTGTTAAGGCACGATAATC‑AATAGGAGAATTAAATATGATCGTTTCTGACATCGAAGCTAACGCCCTCTTATAGAGCTTCACTAAGTGCCACTGCGGGGTTATCTACGACTACTCCACCGCTGAGTACGTAAGCTACCGTCCGAGTGACTGCGTTGCGTATCTGGATGCGCTGGAAGCCGAGGTTGCACGAGGCGGTCTTATTG                                                                           <  1:317181/288‑1`  
                                                                                                                                                                                                                                       `gcgtcatggtGAAGAAGAGTCACCCTCAGAGTCAACAGACTGGTCCAACGACACTGACTTCTGACAGTAATCTTGACAGTTGTGTCATATGACGAGATTTTTAAGTCACGATAATC‑AATAGGAGAAATCAATATGATCGTTTCTGACATCGAAGCTAACGCCCTCTTAGAGAGCGTCACTAAGTTCCACTGCGGGGTTATCTACGACTACTCCACCGCTGATTACGTAAGCTACCGTCCGAGTGACTTCGGTGCGTATTCGGACGTTCTGGAAGCCGAGGTTGCACGAGGCGGTCTTATTG                                                                           <  1:285712/291‑1`  
                                                                                                                                                                                                                                       `ctcggagatgtgtataagagacagCTCAGAGTCAACAGACTGGTCCAACGACACTGACTTCTGACAGGATTCTTGACAGTTGTTTCATATGAAGAGATTGTTAAGTCACGATAATC‑AATAGGAGAAATCAATATGATCGTTTCTGACATCGAAGCTAACGCCCTCTTAGAGAGCGTCACTAAGTTCCACTGCGGGGTTATCTACGACTACTCCACCGCTGAGTACGTAAGCTACCGTCCGAGTGACTTCGGTGCGTATCTGGATGCGCTTGAAGCCGAGGTTGCACGAGGCGGTCTTATTG                                                                           <  1:300844/277‑1`  
                                                                                                                                                                                                                                       `TTACTCCGGGGAAGAAGAGGTACACTCAGAGTCAACAGACTGGTCCAACGACACTGACTTCTGACAGGATTCTTGACAGTTGTTTCATATGAAGACATTGTTAAGTCACGATAATC‑AATAGGAGAAATCAATATGATCGTTTCTGACATCGAAGCTAACGCCCTCTTAGAGAGCGTCACTAAGTTCCACTGCGGGGTTATCTACGACTACTCCACCGCTGAGTACGTAAGCGACCGTCCGAGTGACTTCGGTGCGTATCTGGATGCGCTGGAAGCCGAGGTTGCACGAGGCGGTCTTATTG                                                                           <  1:45385/301‑1`  
                                                                                                                                                                                                                                       `TTACTCAGGGGAAGAAGAGTCACACTCAGAGTCAACAGACTGGTCCAACGACACTGACTTCTGACAGGATTCTTGACAGTTGTTTCATATGAAGAGATTGTTAAGTCACGATAATC‑AATAGGAGAAATCAATATGATCGTTTCTGACATCGAAGCTAACGCCCTCTTAGAGAGCGTCACTAAGTTCCACTGCGGGGTTATCTACGACTACTCCACCGCTGAGTACGTAAGCTACCGTCCGAGTGACTTCGGTGCGTATCTGGATGCGCTGGAAGCCGAGGTTGCACGAGGCGGTCTTATTG                                                                           <  1:224758/301‑1`  
                                                                                                                                                                                                                                       `TTACTCAGGGGAAGAAGAGTCACACTCAGAGTCAACAGACTGGTCCAAAGACACTGACTTCTGACAGGATTCTTGACAGTTGTTTCATATGAAGAGATTGTTAAGTCACGATAATC‑AATAGGAGAAATCAATATGATCGTTTCTGACATCGAAGCTAACGCCCTCTTAGAGAGCGTCACTAAGTTCCACTGCGGGGTTATCTACGACTACTCCACCGCTGAGTACGTAAGCTACCGTCCGAGTGACTTCGGTGCGTATCTGGATGCGCTGGAAGCCGAGGTTGCACGAGGCGGTCTTATTG                                                                           <  1:322086/301‑1`  
                                                                                                                                                                                                                                         `ACTCAGGGGAAGAAGAGTCACACTCAGAGTCAACAGACTGGTCCAACGACACTGACTTCTGACAGGATTCTTGACAGTTGTTTCATATGAAGAGATTGTTAAGTCACGATAATCAAACAGGAGAAACAATTATGATCGTTTCTGACATCGAAGCTAACGCCCTCTTAGAGAGCGTCACTAAGTTCCACTGCGGGGTTATCTACGACTACTCCACCGCTGAGTACGTAAGCTACCGTCCGAGTGACTTCGGTGCGTATCTGGATGCGATGGAAGCCGAGGTTGCACGAGGCGGTCTTCTTGT                                                                          >  1:401666/1‑301`  
                                                                                                                                                                                                                                         `ACTCAGGGGAAGAAGAGTCACACTCAGAGTCAACAGACTGGTCCAACGACACTGACTTCTGACAGGATTCTTGACAGTTGTTTCATATGAAGAGATTGTTAAGTCACGATAATC‑AATAGGAGAAATCAATATGATCGTTTCTGACATCGAAGCTAACGCCCTCTTAGAGAGCGTCACTAAGTTCCACTGCGGGGTTATCTACGACTACTCCACCGCTGAGTACGTAAGCTACCGTCCGAGTGACTTCGGTGCGTATCTGGATGCGCTGGAAGCCGAGGTTGCACGAGGCGGTCTTATTGTG                                                                         >  1:450182/1‑301`  
                                                                                                                                                                                                                                            `acaggtAAGAAGATTCACACTGATAGTCAGCAGACTGGTCCCACGACATTGATTTCTGTCAGGATTATTGACATTTGTTTAATATGAAGAGATTGTTAAGTCACGTTAATC‑AATAGGAGAAATCAATATGATCGTTTCTGACATCGAAGCTAACGCCCTCTTAGATAGCGTCACTAAGTTCCACTGCGGGGTTATCTACGACTACTCCACCGCTGAGTACGTAAGCTACCGTCCGAGTGACTTCGGTGCGTATCTGGATGCGCTGGAAGCCGAGGTTGCACGAGGCGGTCTTATTGTGTTC                                                                      <  1:67999/295‑1`  
                                                                                                                                                                                                                                            `CAGGGGAAGAAGAGTCACACTCGGAGTCAACAGACTGGTCCAACGACACTGACTTCTGACAGGATTCTTGACAGTTGTTTCATATGAAGAGATTGTTAAGTCACGATAATC‑AATAGGAGAAATCAATATGATCGTTTCTGACATCGAAGCTAACGCCCTCTTAGAGAGCGTCACTAAGTTCCACTGCGGGGTTATCTACGACTACTCCACCGCTGAGTACGTAAGCTACCGTCCGAGTGACTTCGGTGCGTATCTGGATGCGCTGGAAGCCGAGGTTGCACGAGGCGGTCTTATTGTGTTC                                                                      <  1:456790/301‑1`  
                                                                                                                                                                                                                                              `GGGGAAGAAGAGTCACACTCAGAGTCAACAGACTGGTCCAACGACACTGACTTCTGACAGGATTCTTGACAGTTGTTTCATATGAAGAGATTGTTAAGTCACGATAATC‑AATAGGAGAAATCAATATGATCGTTTCTGACATCGAAGCTAACGCCCTCTTAGAGAGCGTCCCTAAGTTCAACTGCGGGGTTATCTACGACTACTCCACCGCTGAGTACGTAAGCTACCGTCCGAGTGACTTCGGTGCGTATCTGGATGCGCTGGAAGCCGAGGTTGCACGAGGCGGTCTTATTGctgtctc                                                                    >  1:503462/1‑294`  
                                                                                                                                                                                                                                              `GGGGAAGAAGAGTCACACTCAGAGTCAACAGACTGGTCCAACGACACTGACTTCTGACAGGATTCTTGACAGTTGTTTCATATGAAGAGATTGTTAAGTCACGATAATC‑AATAGGAGAAATCAATATGATCGTTTCTGACATCGAAGCTAACGCCCTCTTAGAGAGCGTCACTAAGTTCCACTGCGGGGTTATCTACGCCTACTCCACCTCTGCGTACGTAAGCTACCGTCCGAGTGACTTCGTTGCGTATCTGGATGCGCTGGAAGCCGAGGTTGCACGAGGCAGTCTTATTGTGTTCCA                                                                    >  1:113259/1‑301`  
                                                                                                                                                                                                                                              `GGGGAAGAAGAGTCACACTCAGAATCAACAGACTGGTCCAACGACACTGACTTCTGACAGGATTCTTGACAGTTGTTTCATATGAAGAGATTGTTAAGTCACGATAATC‑AATAGGAGAAATCAATATGATCGTTTCTGACATCGAAGCTAACGCCCTCTTAGAGAGCGTCACTAAGTTCCACTGCGGGGTTATCTACGACTACTCCACCGCTCAGTACGTAAGCTACCGTCCGAGTGACTTCGGTGCGTCTCTGGATGCGCTGGAAGCCGAGGTTGCACGAGGCGGTCTTATTGTGTTCCA                                                                    >  1:214170/1‑301`  
                                                                                                                                                                                                                                                 `tataagaaacagCACTCAGAGTCAACAGACTGGTCCAGCGACACTGACTTCTGACAGGATTCTTGACAGTTGTTTCATATGAAGAGATTGTTAAGTAACGATAATC‑AATAGTAGAAAGCAATATGATCGTTTCTGACATCGAAGCTAACGCCCTCTTAGAGAGCGTTACTAAGTTCCACTGCGGGGTTATCTACGACTACTCCACCGCTGAGTACGTAAGCTACCGTCCGAGTGACTTCGGTGCGTATCTGGATGCGCTGGAAGCCGAGGTTGCACGAGGCGGTCTTATTGTGTTCCACAA                                                                 <  1:461182/289‑1`  
                                                                                                                                                                                                                                                               `CTCAGAGTCAACAGACTGGTCCAACGACACTGACTTCTGACAGGATTCTTTACAGTTGTTTCATATGAAGAGATTGTTAAGTCACGATAATC‑AATAGGAGAAATAAATATGATCGTTGCTGACATCGAAGCTAACGCCCTCTTAGAGAGCGTCACTAAGTTCCACTGCGGGGTTATCTACGACTACTCCACCGCTGAGTACGTAAGCAACCGTCAGAGTGACTTCGGTGCGTATCTGGATGCGCTGGAAACTGAGGTTGCAAGAGGCGGGCTTATTGGTTTCCACAACGGTAACAAGTtat                                                   >  1:37728/1‑298`  
                                                                                                                                                                                                                                                               `CTCAGAGTCAACAGACTGGTCCAACGACACTGACTTCTGACAGGATTCTTGACAGTTGTTTCATATGAAGAGATTGTTAAGTCACGATAATC‑AATAGGAGAAATCAATATGATAGTTTCTGACATCGAAGCTAACGCCCTCTTAGAGAGCGTCACTAAGTTCCACTGCGGGGTTATCAACGACTACTCCACCGCTGAGTACGTAAGCTACCGTCCGAGTGACTTCGGTGCGTATATGGATGCGCTGGAAGCCGAGGTTGCACGAGGCGGTCTTATTGTGTTCAACAACGGTCAAAAGTATG                                                   >  1:126376/1‑301`  
                                                                                                                                                                                                                                                                `cCAGAGTCAACAGACTGGTCCAACGACACTGACTTCTGACAGGATTCTTGACAGTTGTTTCATATGAAGAGATTGTTAAGTCACGATAATC‑AATAGGAGAAATCAATATGATCGTTTCTGACATCGAAGCTAACGCCCTCTTAGAGAGCGTCACTAAGTTCCACTGCGGGGTTATCTACGACTACTCCACCGCTGAGTACGTAAGCTACCGTCCGAGTGACTTCGGTGCGTATCTGGATGCGCTGGAAGCCGAGGTTGCACGAGGCGGTCTTATTGTGTTCCACAACGGTCACAAGTATGA                                                  <  1:143058/300‑1`  
                                                                                                                                                                                                                                                                `agAGAGTCAACAGACTGGTCCAACGACACTGACTTCTGACAGGATTCTTGACAGTTGTTTCATATGAAGAGATTGTGAAGTCACGATAATC‑AATAGGAGAAATCAATATGATCGTTTCTGACATCGAAGCTAACGCCCTCTTAGAGAGCGTCACTAAGTTCCACTGCGGGGTTATCTACGACTACTCCACCGCTGAGTACGTAAGCTACCGTCCGAGTGACTTCGGTGCGTATCTGGATGCGCTGGAAGCCGAGGTTGCACGAGGCGGTCTTATTGTGTTCCACAACGGTCACAAGTATGA                                                  <  1:362769/299‑1`  
                                                                                                                                                                                                                                                                   `gtgtataagagacaGTCCAACGACACTGACTTCTGACAGGATTCTTGACAGTTGTTTCATATGAAGAGATTGTTAAGTCACGATAATC‑AATAGGAGAAATCAATATGATCGTTTCTGACATCGAAGCTAACGCCCTCTTAGAGAGCGTCACTAAGTTCCACTGTGGGGTTATCTACGACTACTCCACCGCTGAGTACGTAAGCTACCGTCCGAGTGACTTCGGTGCGTATCTGGATGCGCTGGAAGCCGAGGTTGCACGAGGCGGTCTTATTGTGTTCCACAACGGTCACAAGTATGACGT                                               <  1:240838/287‑1`  
                                                                                                                                                                                                                                                                   `GAGTCAACAGACTGGTCCAACGACACTGACTTCTGACAGGATTCTTGACAGTTGTTTCATATGAAGAGATTGTTAAGTCACGATAATC‑AATAGGAGAAATCAATATGATCGTTTCTGACATCGAAGCTAACGCCCTCTTAGAGAGCGTCACTAAGTTCCACTGCGGGGTTATCTACGACTACTCCACCGCTGAGTATGTAAGCTACCGTCCGAGTGACTTCGGTGCGTATCTGGATGCGCTGGAAGCCGAGGTTGCACGAGGCGGTCTTATTGTGTTCCACAACGGTCACAAGTATGACGT                                               <  1:29525/301‑1`  
                                                                                                                                                                                                                                                                       `CAACAGACTTGTCCAACGACACTGACTTCTGACGGGATTCTTGACAGTTGTTTCATATGAAGAGATTGTTAAGTCACGATAATC‑AATAGGAGAAATCAATATGATCGTTTCTGACATCGAAGCTAACGCCCTCTTAGAGAGCGTCACTAAGTTCCACTGCGGGGTTATCTACGACTACTCCACCGCTGAGTACGTAAGCTACCGTCCGAGTGACTTCGGTGCGTATCTGGATGCGCTGGAAGCCGAGGTTGCACGAGGCGGTCTTATTGTGTTCCACAACGGTCACAAGTATGACGTTCCT                                           <  1:389381/301‑1`  
                                                                                                                                                                                                                                                                       `CAACAGACTGGTCCAACGACACTGACTTCTGACAGGATTCTTGACAGTTGTTTCATATGAAGAGCTTGTTAATTCACGATAATC‑AATAGGAGAAATCAATATGATCGTTTCTGACATCGAAGCTAACGCCCTCTTAGAGAGAGTCTCTAAGTTCCACTGCGTGGTTATCTACGACTACTCCACCGCTGAGTACGTAAGCTACCGTCCGAGTGACTTCGTTTCGTTTCGGGATGCGCTGGAAGCCGAGGTTGCACGAGGCGGTCTTATTGTGCTCCACAACTGCCACACCTATGACTTTCCT                                           >  1:279372/1‑301`  
                                                                                                                                                                                                                                                                        `agatacagGGTCCAACGACACGGACTTCTGACAGGTTTCTGGACAGTTGTTTCATATGAATAGATTGTCAAGTCACGATAATC‑AATAGGAGAAATCAATATGATCGTTTCTGACAGCGAAGCTAACGCCCTCTTAGAGAGCGTCACTAAGTTCCACTGCGGGGTTATCTACGACTACTCCACCGCTGAGTACGTAAGCTACCGTCCGAGTGACTTCGGTGCGTATCTGGATGCGCTGGAAGCCGAGGTTGCACGAGGCGGTCTTATTGTGTTCCACAACGGTCACAAGTATGACGTTCCTG                                          <  1:62053/293‑1`  
                                                                                                                                                                                                                                                                           `AGACTGGTCCAAATACAATGACGTCTGACAGGATACTTGACAGTTGTGTCATATGAAGAGATTGTGAAGTCACGATAATC‑AATAGGAGAAATCAATATGATAGTTTCTGACAGCGAAGCTAACGCCCTCTTAGAGAGCGTCACTAAGTGCCACTGCGGGGTTATCTACGACGACGCCACCGCTGAGTACGTAAGCAACCGTCCGAGTGACTTCGGTGTGTATCTGGATGCGCTGGAAGCCGAGGTTGCAAGAGGCGGTCTTATTGTGTTCCACAACGGTCACAAGTATGACGTTCCTGCAT                                       <  1:64817/301‑1`  
                                                                                                                                                                                                                                                                            `GACTGGTCCAACGACACTGACTTCTGACAGGATTCTTGACAGTTGTTTCATATGAAGAGATTGTTAAGTCACGATAATC‑AATAGGAGAAATCAATATGATCGTTTCTGACATCGAAGCTAACGCCCTCTTAGAGAGCGTCACTAAGTTCCACTGCGGGGTTATCTACGACTACTCCACCGCTGAGTACGTAAGCTACCGTCCGCGTGACTTCGGTGCGTATCTGGATGCGCTGGAAGCCGAGGTTGCACGAGGCGGTCTTATTGTGTTCCACAACGGTCACAAGTATGACGTTCCTGCATT                                      >  1:258333/1‑301`  
                                                                                                                                                                                                                                                                                `GGTCCAACGACACTGACTTCTGACAGGATTCTTGACAGTTGTTTCATATGAAGAGATTGTTAAGTCACGATAATC‑AATAGGAGAAATCAATATGATCGTTTCTGACATCGAAGCTAACGCCCTCTTAGAGAGCGTCACTAAGTTCCACTGCGGGGTTATCTACGACTACTCCACCGCTGAGTACGTAAGCTACCGTCCGAGTGACTTCGGTGCGTATCTGGATGCGCTGGAAGCCGAGGTTGCACGAGGCGGCCTTATTGTGTTCCACAACGGTCACAAGTATGACtgtctcttatcccca                                  >  1:291007/1‑286`  
                                                                                                                                                                                                                                                                                `GGTCCAACGACACTGACTTCTGACAGGATTCTTGACAGTTGTTTCATATGAAGAGATTGTTAAGTCACGATAATC‑AATAGGAGAAATCAATATGATCGTTTATGACATCGAAGCTAACGCCCTCTTAGAGAGCGTCACTAAGTTCCACTGCGGGGTTATCTACGACTACTCCACCGCTGAGTACGTAAGCTACCGTCCGAGTGACTTCGGTGCGTATCTGGATGCGCTGGAAGCCGAGGTTGCACGAGGCGGTCTTCTTGTGTTCCACAACGGTCACAAGTATGAAGTTCCTGCATTGACC                                  >  1:115733/1‑301`  
                                                                                                                                                                                                                                                                                         `ACAATGACTTCTGACAGGATTCTTGACAGTTGTTTCATATGAAGAGATTGTTAAGTCACGATAATC‑AATAGGAGAAATCAATATGATCGTTTCTGACATCGAAGCTAACGCCCTCTTAGAGAGCGTCACTAAGTTCCACTGCGGGGTTATCTACGACTACTCCACCGCTGAGTACGTAAGCTACCGTCAGAGTGACTTCGGTGCGTATCTGGATGCGCTGGAAGCCGAGGTTGCACGAGGCGGTCTTATTGTGTTCCACAACGGTAACAAGTATGACGTTCCTGCATTGACCAAACTGGCA                         <  1:10175/301‑1`  
                                                                                                                                                                                                                                                                                          `CACTGAATTCTGACAGGATTCTTGACAGTTGTTTCATATGAAGAGATTGTTAAGTCACGATAATC‑AATAGGAGAAATCAATATGATCGTTTCTGACATCGAAGCTAACGCCCTCTTAGAGAGCGTCACTAAGTTCCACTGCGGGGTTATCTACGACTACTCCACCGCTGAGTACGTAAGCTACCGTCCGAGTGACTTCGGTGCGTATCTGGATGCGCTGGAAGCCGAGGTTGCACGAGGCGGTCTTATTGTGTTCCACAACGGTCACAAGTATGACGTTCCTGCATTGACCAAACTGGCAA                        <  1:433631/301‑1`  
                                                                                                                                                                                                                                                                                           `accGACTTCTGACAGGATTCTTGACAGTTGTTTCATATGAAGAGATTGTTAAGTCACGATAATC‑AATAGGAGAAATCAATATGATCGTTTCTGACATCGAAGCTAACGCCCTCTTAGAGAGCGTCACTAAGTTCCACTGCGGGGTTATCTACGACTACTCCACCGCTGAGTACGTAAGCTACCGTCCGAGTGACTTAGGTGCGTATCTGGATGCGCTGGAAGCCGAGGTTGCACGAGGCGGTCTTATTGTGTTCCACAACGGTCACAAGTATGACGTTCCTGCATTGACCctgtctcttat                       >  1:187116/4‑290`  
                                                                                                                                                                                                                                                                                              `GACTTCTGACAGGATTCTTGACAGTTGTTTCATATGAAGAGATTGTTAAGTCACGATAATC‑AATAGGAGAAATCAATATGATCGTTTCTGACATCGAAGCTAACGCCCTCTTAGAGAGCGTCACTAAGTTCCACTGCGGGGTTATCTACGACTACTCCACCGCTGAGTACGTAAGCTACCGTCCGAGTGACTTCGGTGCGTATCTGGATGCGCTGGAATCCGAGGTTGCACGAGGCGGTCTTATTGTGTTCCACAACGGTCACAAGTATGACGctgtctcttatacacatctccgagcccc                    >  1:215879/1‑273`  
                                                                                                                                                                                                                                                                                                `CTTCTGACAGGATTCTTGACAGTTGTTTCATATGAAGAGATTGTTAAGTCACGATAATC‑AATAGGAGAAATCAATATGATCGTTTCTGACATCGAAGCTAACGCCCTCTTAGAGAGCGTCACTAAGTTCCACTGCGGGGTTATCTACGACTACTCCACCGCTGAGTACGTAAGCTACCGTCCGAGTGACTTCGTTGCGTATCTGGATGCGCTGGAAGCCGAGGTTGCACGAGGCGGTCTTATTGTGTTCCACAACGGTCACAAGTATGACGTTCCTGCATTGACCAAACTGGCAAAGTTGC                  >  1:358686/1‑301`  
                                                                                                                                                                                                                                                                                                     `GACAGGATTCTTGACAGTTGTTTCATATGAAGAGATTGTTAAGTCACGATAATC‑AATAGGAGAAATCAATATGATCGTTTCTGACATCGAAGCTAACGCCCTCTTAGAGAGCGTCACTAAGTTCCACTGCGGGGTTATCTACGACTACTCTACCGCTGAGTACGTAAGCTACAGTCCGAGTGACTTCGGTGCGTATCTGGATGCGCTGGAAGCCGAGGTTGCACGCGGCGGTCTTATTGTGTTCCAAAACGGTCACAAGTATGACGTTCCTGCATTTACCAAACTGGCAAAGTTGCAATTG             >  1:467139/1‑301`  
                                                                                                                                                                                                                                                                                                           `ATTCTTGACAGTTGTTTCATATGAAGAGATTGTTAAGTCACGCTAATC‑AAAAGGAGAAATTAATATGATAGTTTCTGGCATCGAATCTAACGCCCTCTTAGAGAGCGTCACTAAATTCTACTGCGGGGTTATCTAAGACCACTACACTGCTGAGTACGTAAGCTACCGTCCGATAGGCTTCGGTTCGCATCTGGATGCGCTGGAAGCCGACGTTGAACGAAGCGGTCGTATTTTGTTCCACAACGGTCACATGTATAACGTTCATGCATTAACCACACTGGCAACGTTGCAATTGACCCta       >  1:508285/1‑299`  

                                                                                                                                                                                                                                                                                                                                                                                                                                                                                                                                                                                                                                
`AGACAAAGGTAAAGCACATGAGGAAGGTCGCCCCGTTTCTATTACTGACCTACGTGGTTCTGGCGCACTACGCCAACTATCTGATACTATTATTGCCCTTGAGCGTAATCAGCAAGGCGATATGCCTAACCTTGTCCTCGTTCGTATTCTCAAGTGCCGCTTTACTGGTGATACTGGTATCGCTGGCTACATGGAATACAACAAGGAAACCGGATGGCTTGAACCATCAAGTTACTCAGGGGAAGAAGAGTCACACTCAGAGTCAACAGACTGGTCCAACGACACTGACTTCTGACAGGATTCTTGA‑‑‑‑‑‑‑‑‑‑‑‑‑‑‑‑‑‑‑‑‑‑‑‑‑‑‑‑‑‑‑‑‑‑‑‑‑‑‑‑‑‑‑‑‑‑‑‑‑‑‑‑‑‑‑‑‑‑‑‑‑‑‑‑‑‑‑‑‑‑‑‑‑‑‑‑‑‑‑‑‑‑‑‑‑‑‑‑‑‑‑‑‑‑‑‑‑‑‑‑‑‑‑‑‑‑‑‑‑‑‑‑‑‑‑‑‑‑‑‑‑‑‑‑‑‑‑‑‑‑‑‑‑‑‑‑‑‑‑‑‑‑‑‑‑‑‑‑‑‑‑‑‑‑‑‑‑‑‑‑‑‑‑‑‑‑‑‑‑‑‑‑‑‑‑‑‑‑‑‑‑‑‑‑‑‑‑‑‑‑‑‑‑‑‑‑‑‑‑‑‑‑‑‑‑‑‑‑‑‑‑‑‑‑‑‑‑‑‑‑‑‑‑‑‑‑‑‑‑‑‑‑‑‑‑‑‑‑‑‑‑‑‑‑‑‑‑‑‑‑‑‑‑‑‑‑‑‑‑‑‑‑‑‑‑‑‑‑‑‑‑‑‑‑‑‑‑‑‑‑‑‑‑‑‑‑‑‑‑‑‑‑‑‑‑‑‑‑‑  >  NC_001604.1/12971‑13277`  
`‑‑‑‑‑‑‑‑‑‑‑‑‑‑‑‑‑‑‑‑‑‑‑‑‑‑‑‑‑‑‑‑‑‑‑‑‑‑‑‑‑‑‑‑‑‑‑‑‑‑‑‑‑‑‑‑‑‑‑‑‑‑‑‑‑‑‑‑‑‑‑‑‑‑‑‑‑‑‑‑‑‑‑‑‑‑‑‑‑‑‑‑‑‑‑‑‑‑‑‑‑‑‑‑‑‑‑‑‑‑‑‑‑‑‑‑‑‑‑‑‑‑‑‑‑‑‑‑‑‑‑‑‑‑‑‑‑‑‑‑‑‑‑‑‑‑‑‑‑‑‑‑‑‑‑‑‑‑‑‑‑‑‑‑‑‑‑‑‑‑‑‑‑‑‑‑‑‑‑‑‑‑‑‑‑‑‑‑‑‑‑‑‑‑‑‑‑‑‑‑‑‑‑‑‑‑‑‑‑‑‑‑‑‑‑‑‑‑‑‑‑‑‑‑‑‑‑‑‑‑‑‑‑‑‑‑‑‑‑‑‑‑‑‑‑‑‑‑‑‑‑‑‑‑‑‑‑‑‑‑‑‑‑‑‑‑‑‑‑‑‑‑‑‑‑‑‑‑‑‑‑‑‑‑‑‑‑‑‑‑‑‑‑‑‑‑‑‑‑‑ttcttgaCAGTTGTTTCATATGAAGAGATTGTTAAGTCACGATAATC‑AATAGGAGAAATCAATATGATCGTTTCTGACATCGAAGCTAACGCCCTCTTAGAGAGCGTCACTAAGTTCCACTGCGGGGTTATCTACGACTACTCCACCGCTGAGTACGTAAGCTACCGTCCGAGTGACTTCGGTGCGTATCTGGATGCGCTGGAAGCCGAGGTTGCACGAGGCGGTCTTATTGTGTTCCACAACGGTCACAAGTATGACGTTCCTGCATTGACCAAACTGGCAAAGTTGCAATTGAACCGAGAGTT  >  NC_001604.1/14297‑14594`

  

Alignment Legend

Aligned base mismatch/match (shaded by quality score): `ATCG/ATCG` `< 3 ≤` `ATCG/ATCG` `< 10 ≤` `ATCG/ATCG` `< 20 ≤` `ATCG/ATCG` `< 34 ≤` `ATCG/ATCG` `< 38 ≤` `ATCG/ATCG`

Unaligned base: `atcg`    Masked matching base: `atcg`    Alignment gap: `‑`    Deleted base: `‑`

Reads not counted as support for junction

read\_name Not counted due to insufficient overlap past the breakpoint.

read\_name Not counted due to not crossing MOB target site duplication.
